# Navigating lymphatic drainage uncertainty in abdominal ectopic breast cancer: a novel application of indocyanine green fluorescence—a case report and literature review

**DOI:** 10.1007/s13691-026-00910-1

**Published:** 2026-09-04

**Authors:** Alessio Vinci, Nadia Khitab, Hasan Asfour, Vassilis Pitsinis

**Affiliations:** 1 Breast Surgery unit, Ninewells Hospital, NHS Tayside, Dundee, UK; 2 University of Dundee Medical School, Dundee, UK; 3 Leicester Cancer Research Centre, The School of Medical Sciences, University of Leicester Clinical Sciences Building Leicester Royal Infirmary Leicester, LE1 5WW Leicester, UK

**Keywords:** Ectopic breast, Breast cancer, ICG, lymphatic Mapping, SLNB, Case report

## Abstract

Ectopic breast cancer (EBC) is a rare condition in which lymphatic drainage mapping can be challenging due to its variable anatomical locations along the milk line, extending from the axilla to the groin. The EBC arising from the abdominal wall represents a unique challenge, as lymphatic drainage is unpredictable and may involve the axillary and/or inguinal nodal basins. Indocyanine green (ICG) fluorescence has recently emerged as a reliable tracer guiding sentinel lymph node biopsy (SLNB) in orthotopic breast cancer; however, its use in EBC has not yet been reported. Hereby, we report a case of a 64-year-old postmenopausal Caucasian woman who presented with a progressively enlarging mass arising from an ectopic breast tissue in the left upper anterior abdominal wall. Core biopsy revealed invasive papillary carcinoma of breast tissue with associated ductal carcinoma in situ. The patient underwent excision of the ectopic breast tissue involving the EBC with SLNB using ICG fluorescence as the sole tracer. Real-time fluorescence imaging demonstrated lymphatic drainage to the ipsilateral axilla with no inguinal drainage identified. One axillary sentinel lymph node was excised and was negative for metastasis. At one-year follow-up, there was no evidence of recurrence or distant metastasis. To our knowledge, this is the first report describing the use of ICG fluorescence for lymphatic mapping in EBC to guide SLNB. The ability to visualize lymphatic drainage intraoperatively was particularly valuable given the aberrant tumor location and the potential for variable lymphatic drainage pathways. We also reviewed the existing literature on cases of EBC arising from abdominal wall, highlighting the variability in lymphatic drainage patterns and the inconsistent use or underreporting of lymphatic mapping techniques and SLNB, resulting in limited understanding of nodal staging and potential implications for management strategies.

## Introduction

Ectopic breast tissue (EBT) occurs due to incomplete embryologic regression of the mammary ridges (milk lines). The most common location of EBT is the axilla; however, it can occur anywhere along the milk line, which extends bilaterally from the axilla to the groin [1]. The EBT may also occur, although extremely rare, outside of the milk line, such as in the upper extremities or the back [2, 3]. The prevalence of EBT has been estimated at up to 6% among the female population, with a relatively lower prevalence among men [4].

The EBT is vulnerable to the same pathologies that affect orthotopic breast tissue, notably cancer. Ectopic breast cancer (EBC) accounts for 0.3–0.6% of all breast cancers [5], with majority of cases occurring in the axilla, while abdominal wall EBC is exceedingly rare [6]. Abdominal wall-arising EBC represents a unique clinical challenge, as lymphatic drainage is unpredictable and may involve the axillary and/or inguinal nodal basins; however, reported cases remain scarce, with limited and inconsistent reporting of lymphatic mapping and SLNB, contributing to uncertainty of nodal staging and suboptimal management [6–9].

The process of lymphatic drainage mapping for nodal staging in EBC is similar to that used in orthotopic breast cancer [10]. During sentinel lymph node biopsy (SLNB), common tracing agents include radioactive isotopes (e.g., technetium-99 m) alone or combined with blue dye (e.g., methylene blue and isosulfan blue) are used [11, 12]. Although the use of both radioactive isotopes and blue dye has traditionally been considered highly effective, they carry certain limitations including nuclear hazards associated with radioisotopes and risk of allergic reaction and infection, and lower detection rates with blue dye [13–16].

The use of indocyanine green (ICG) fluorescent dye has recently emerged as an alternative to conventional tracers and it has generally been associated with comparable detection rates, advantageous intraoperative visibility, and lower costs [17–20]. However, there are currently no reports regarding the use of ICG, particularly as a single tracer, in SLNB for patients with EBC. To address this knowledge gap, we hereby present the first consistent case report of lymphatic drainage mapping and SLNB using ICG solely in a patient with abdominal EBC, demonstrating its safety and reliability in this context. We also reviewed the existing literature on cases of EBC arising from the anterior abdominal wall, highlighting the variability in lymphatic drainage patterns and the inconsistent use of lymphatic mapping techniques and SLNB, resulting in limited understanding of nodal staging and potential implications for management strategies (Table 1).

## Case presentation

A 64-year-old post-menopausal Caucasian female presented with a 9-month history of an irregular lump in the left upper anterior abdominal wall. The lump was first noticed by the patient during a shower due to mild pain when rubbed. The mass progressively increased in size and started to protrude, causing discomfort as it rubbed against the patient’s clothing. The patient was referred to the breast unit after plastic surgery ruled out sarcoma. Clinical examination was consistent with a suspicious lump in an EBT (polymastia). The lump was mobile, well-circumscribed, and not attached to the skin, costal margin, or underlying structures. Further diagnostic planning was recommended following a multidisciplinary discussion to inform an appropriate management strategy.

An ultrasound scan of the abdominal wall was performed and showed a 3 cm-sized ill-defined mass, while the orthotopic breast tissue of both breasts was unremarkable. Bilateral mammography was then arranged, which was unremarkable. There was also no evidence of lymphadenopathy in the groin and the axillae. Ultrasound-guided core-biopsy revealed a grade 2 invasive mammary cancer (ER8, PR5, HER2 Negative) with an intermediate-grade ductal carcinoma in situ (DCIS).

**Fig. 1:**
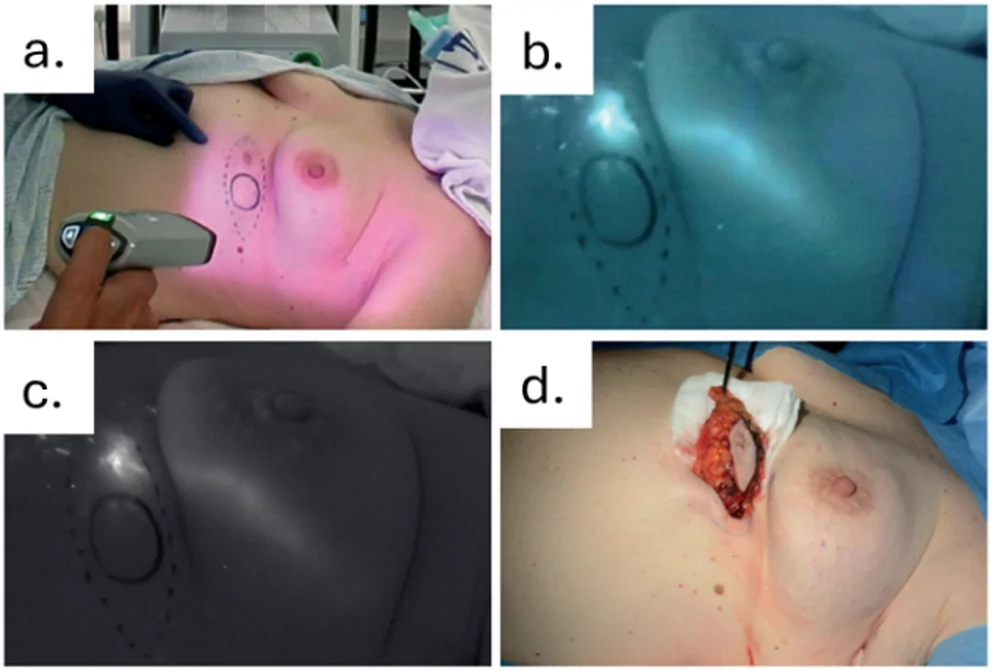
Preparation for complete excision of the abdominal ectopic breast tissue involving the cancer (a) with lymphatic nodal mapping using Indocyanine green only (**b**,** c**) prior to complete excision of the ectopic breast tissue (**d**)

**Fig. 2:**
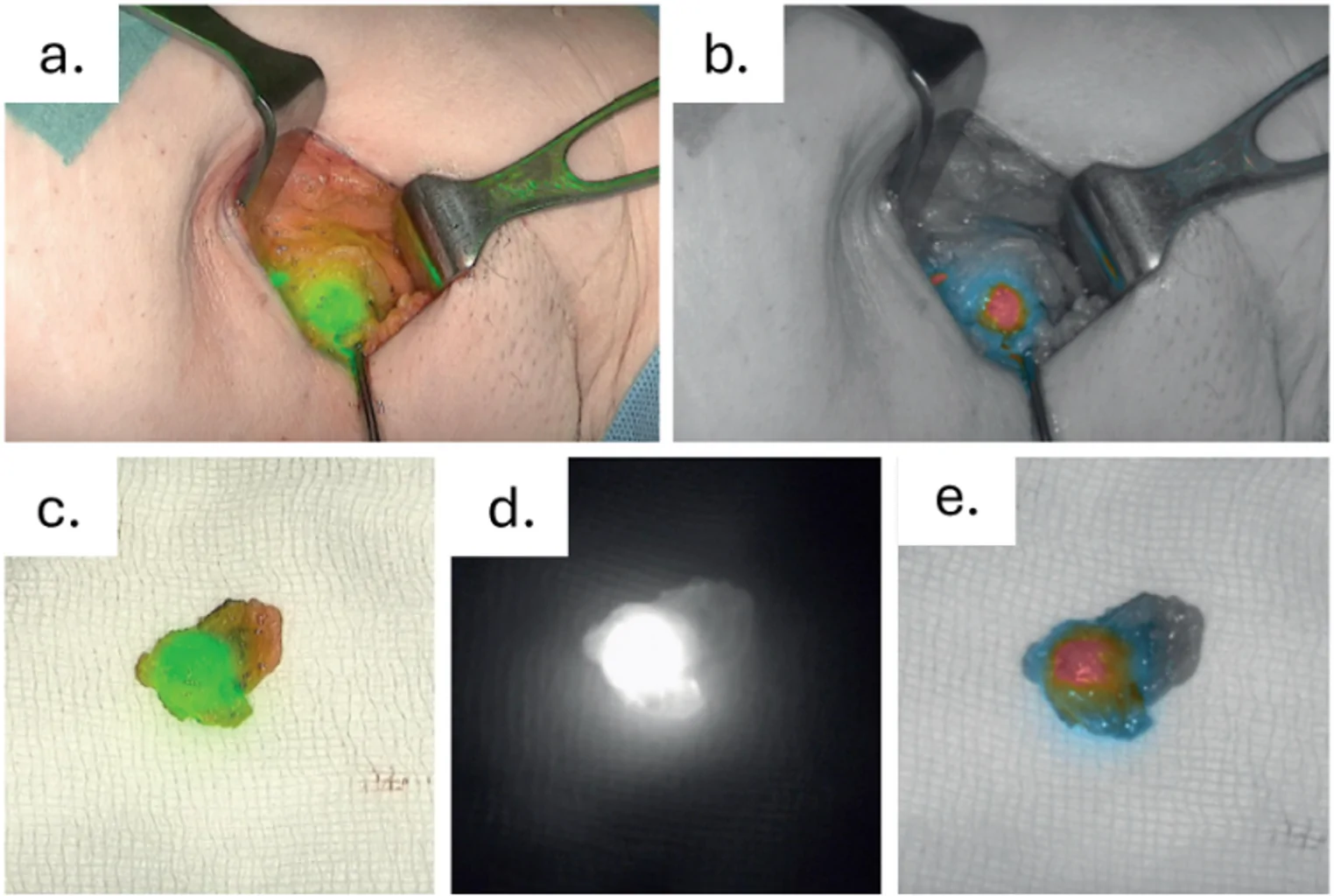
Nodal retrieval using the Indocyanine green-only technique before excision, i.e., in vivo (a, b), and after sentinel lymph node excision, i.e., ex vivo (**c**,** d**,** e**)

The patient was offered surgery and planned for full excision of the tumor along with EBT and nodal mapping using ICG solely. Given the atypical anatomical location of EBC and the unpredictable lymphatic drainage, accurate identification of the sentinel lymph node was considered essential for cancer staging. Therefore, nodal mapping was performed using ICG fluorescence imaging as the sole tracer, enabling real-time visualization of lymphatic pathways (Fig. 1). Immediately before surgery, 2 mL of 0.5% indocyanine green (ICG) was injected at the level of the EBT according to our institutional protocol. One milliliter was injected intradermally directly overlying the tumor, and one millilitre was injected subareolar, as nipple-associated complex (NAC) was present in the EBT (Fig. 1A). This technique was selected to mimic the standard lymphatic mapping approach used in orthotopic breast cancer by targeting the superficial lymphatics while allowing real-time visualization of lymphatic drainage.” The fluorescence-guided surgery camera provided real-time visualization of the lymphatic pathway, confirming direct nodal drainage into the ipsilateral axilla (Fig. 2). No drainage into the ipsilateral inguinal area was identified. Axillary sentinel node biopsy was therefore performed along with the EBT excision.

Histopathological testing confirmed the presence of a 30 mm G2 invasive solid papillary carcinoma associated with DCIS (whole tumor diameter was 57 mm). One sentinel node was identified and was negative for metastasis. There was no evidence of Paget’s disease on the nipples.

The patient tolerated the procedure well with no post-operative complications. A five-year regimen of adjuvant Letrozole was planned. At one-year follow-up, the patient remained free of local or regional recurrence on clinical examination. The abdominal surgical site was monitored with ultrasound, which showed no evidence of local recurrence, while bilateral mammography demonstrated no abnormalities within the orthotopic breasts. No regional or distant recurrence was identified during the follow-up period.

## Discussion

The EBT is the most common congenital abnormality of breast development; however, the occurrence of EBC is rare, with most EBCs arising in the axilla [4]. Abdominal wall-arising EBC, as reported in this case, is extremely rare, and only a few cases have been reported in the literature [6]. The rarity of EBC, and abdominal wall EBC in particular, has been associated with considerable delays in diagnosis, likely due to its asymptomatic nature or misdiagnosis, with one comprehensive literature review study reporting, on average, a delay of 40.5 months (range 0 months—20 years) [21]. Therefore, it is essential to ensure prompt diagnosis and management of this condition.

A narrative literature review was conducted using PubMed and Google Scholar to identify published reports of ectopic breast cancer arising from the anterior abdominal wall. The search included articles published up to May 2026 using combinations of the terms ‘ectopic breast cancer’, ‘accessory breast cancer’, ‘abdominal wall’, ‘abdominal ectopic breast’, and ‘supernumerary breast’. As this was a narrative review, no formal systematic review methodology was applied.

Abdominal wall EBC remains exceptionally rare, with only a limited number of reported cases in the literature (Table 1). The available cases demonstrate marked heterogeneity in patient demographics, anatomical site, histology, receptor profile, nodal evaluation, treatment, and outcomes. Reported cases include both female and male patients, with tumor locations ranging from the upper abdomen and umbilicus to suprapubic and inguinal regions (Table 1) [8, 9, 22–24]. Histological subtypes were also variable, including invasive ductal carcinoma, ductal carcinoma in situ (DCIS), invasive lobular carcinoma, inflammatory carcinoma, Paget’s disease, and poorly differentiated carcinoma (Table 1) [7, 23, 25, 26]. Receptor profiles were variable, with the most common profile being hormone receptor–positive and HER2 negative status (Table 1) [8, 9, 24, 25, 27].

The presence of an associated intraductal component (ductal carcinoma in situ) is an important histopathological feature supporting the diagnosis of a primary ectopic breast carcinoma rather than metastatic breast carcinoma or another primary cutaneous adenocarcinoma [28]. In the present case, the invasive papillary carcinoma was associated with intermediate-grade DCIS, supporting its origin within ectopic breast tissue.

The diagnosis and management of abdominal wall EBC generally follow the same principles applied to orthotopic breast cancers; nevertheless, the aberrant anatomical location of breast cancer introduces diagnostic and management challenges, particularly with regard to mapping the lymphatic drainage and nodal staging. Although it may be anatomically more likely that the lymphatic drainage of upper abdominal EBC occurs towards the axilla, like in the current case, it might not be the case in EBCs arising from the lower abdomen, as drainage to the groin is more likely (Table 1) [23–25, 27]. Therefore, the presence of inguinal lymphadenopathy, even if coexisting with orthotopic breast cancer, should raise a high level of suspicion of synchronous or metachronous abdominal or groin EBC draining to inguinal lymph nodes [29].

The use of SLNB and lymphatic mapping was inconsistent across reported cases. Several cases did not undergo SLNB, and lymphatic drainage was either not reported or inferred indirectly from imaging workup or nodal involvement (Table 1). Only a minority of cases included formal nodal assessment. Fachinetti et al. (2018) used radiotracer-based mapping and demonstrated axillary drainage, while the current case used indocyanine green (ICG) fluorescence as a single tracer, confirming ipsilateral axillary drainage without inguinal drainage. These findings support the value of real-time lymphatic mapping in abdominal wall EBC, particularly because drainage may not follow predictable regional patterns.

The sentinel lymph node (SLN) identification using the radioisotopes or blue dye has been shown to be feasible but technically challenging in EBCs, particularly if the tumor exists closer to regional lymphatic basins (e.g. axilla and groin), which may result in a shine-through effect with radioisotopes and diffuse staining when blue dye is used [30, 31]. These reports collectively support the concept that SLN mapping is valuable in EBC, while also emphasizing the need for adaptable, real-time mapping techniques. In this context, ICG fluorescence-guided lymphatic mapping may offer a distinct advantage by enabling dynamic visualization of lymphatic channels, which may mitigate some limitations seen with conventional tracers; however, fluorescence imaging can still be affected by background signal or high-intensity fluorescence at the injection site, although typically less problematic than the shine-through effect of radioisotopes or the diffuse staining observed with blue dye [32].

While the utility of ICG is proven for SLN mapping, much of the available evidence highlighting its benefits associated with the real-time visualization is based on orthotopic breast cancer cases and the use of ICG and another conventional tracer as dual tracers [18, 33, 34]. In this report, we used ICG fluorescence as a single tracer, informed by the findings of the INFLUENCE clinical trial, which established the accuracy of using ICG fluorescence as a standalone lymph node tracer in orthotopic early-stage breast cancers, with no significantly added benefits of combining ICG with another tracer [35]. In addition, the use of ICG is considered safer and technically advantageous compared to other conventional tracers, which are associated with increased costs and the hazards related to the handling of radioactive materials (i.e., isotopes) and the higher risks of allergic reaction, infection, and necrosis associated with blue dye materials [14, 15, 35]. Although lymphoscintigraphy may provide preoperative information regarding lymphatic drainage, particularly in anatomically atypical lesions where both axillary and inguinal drainage are possible, our institutional practice is to perform sentinel lymph node mapping using ICG fluorescence alone. In the present case, real-time fluorescence imaging successfully delineated lymphatic drainage to the ipsilateral axilla without evidence of inguinal drainage, allowing accurate sentinel lymph node biopsy without the need for a radioactive tracer.

Nevertheless, this report has inherent limitations. An ultrasound examination of the abdominal wall, performed as part of the standard triple assessment, Unfortunately, the original ultrasound images and detailed sonographic descriptors (including BI-RADS assessment, vascularity, and posterior acoustic features) were not available for retrospective review. The diagnosis was subsequently established by ultrasound-guided core needle biopsy, which demonstrated invasive mammary carcinoma with associated ductal carcinoma in situ. In addition, as a single case, the findings cannot be generalized, and further studies are required to validate the reliability and reproducibility of the use of ICG as the sole tracer to inform SLNB in EBCs. Additionally, long-term oncological outcomes remain to be established.

In conclusion, the literature highlights abdominal wall EBC as a heterogeneous entity. The inconsistent reporting of lymphatic mapping and SLNB limits understanding of its metastatic pathways and complicates treatment standardization and potentially impacts prognosis. The current case contributes to this evidence gap by demonstrating that ICG fluorescence can safely and effectively define lymphatic drainage intraoperatively and guide SLNB in abdominal wall EBC. This approach may be particularly valuable in cases where drainage to either axillary or inguinal basins is possible.

Summary of cases reporting EBC arising from the anterior abdominal wall.

**Table 1:** Summary of the reported cases of ectopic breast cancer arising from the anterior abdominal wall in literature. This table highlights the variability in lymphatic drainage patterns (axillary versus inguinal), the inconsistent use/underreporting of lymphatic drainage mapping and SLNB, and the heterogeneity in tumor characteristics and management approaches. Abbreviations: F, female; EBC, ectopic breast cancer; SLNB, sentinel lymph node biopsy; SLN, sentinel lymph node; ER, estrogen receptor; PR, progesterone receptor; HER2, human epidermal growth factor receptor 2; DCIS, ductal carcinoma in situ; ICG, indocyanine green

Study	Age/ gender	Cancer location	Lymphatic mapping	Lymphatic drainage	SLNB and SLN status	Nodal/extranodal metastasis	Type of cancer	Receptor status	Treatment	Follow up/ Recurrence
[22] Kopanakis et al. (2016)	58/M	Umbilicus	Not reported	Not reported	Not performed	No nodal or distant metastasis on imaging workup	Poorly differentiated adenocarcinoma	ER and PR: positive, HER2: not reported	Surgical excision (initial + re-excision for margins) → Planned adjuvant chemotherapy and hormonal therapy	No recurrence or metastasis at 10 months
[7] Miles et al. (2017)	58/F	Left upper abdomen (ipsilateral to prior orthotopic breast cancer)	Not reported	Not reported	Performed: SLN negative (one left axillary SLN)	No nodal or distant metastasis on CT/PET; suspicious lympho-vascular invasion.	Inflammatory invasive ductal carcinoma (grade 3) with associated DCIS	ER: positive, PR: negative, HER2: negative	Neoadjuvant chemotherapy → Surgical resection (wide excision) → Radiation therapy with capecitabine → Long-term endocrine therapy (anastrozole)	Resolution of local inflammatory changes after treatment but long-term recurrence was not clearly reported; abdominal EBC lesion occurred 9 years after initial breast cancer
[24] Zhong et al. (2018)	62/M	Right lower abdomen	Not reported	To inguinal and pelvic lymphatic basins, based on imaging findings (axillary basins not assessed)	Not performed	Extensive nodal metastasis: bilateral inguinal, pelvic, retroperitoneal (iliac) lymph nodes; no distant organ metastasis reported	Grade II infiltrating ductal carcinoma with neuro-endocrine features	ER and PR: positive, HER2: negative	Surgical excision → Radiotherapy (abdominal and pelvic regions) → Endocrine therapy (tamoxifen)	Partial response with reduction in lymph node size; stable disease on short-term follow-up
[23] Fachinetti et al. (2018)	76/F	Two metachronous tumors: Right upper abdomen (first tumor) and contralateral (left) upper abdomen (second tumor)	Performed using radiotracer (radioisotope) injection with preoperative lymphoscintigraphy and intraoperative gamma probe	To axillary lymphatic basin (right axilla initially; left axilla for second lesion)	Performed First tumor: SLN negative (one right axillary SLN) Second tumor: SLN positive (left axilla, macro-metastasis)	First tumor: no nodal metastasis; Second tumor: extensive nodal metastasis (11/15 axillary nodes positive); no distant metastasis reported	Invasive lobular carcinoma (both tumors)	First tumor: positive ER and PR, negative Her2; Second tumor: positive ER positive, negative PR, negative Her2	First tumor: Extended excision + SLNB → endocrine therapy (letrozole); Second tumor: Excision → SLNB → axillary lymph node dissection → chemotherapy ± planned radiotherapy (refused)	Metachronous recurrence/new primary at 19 months (contralateral abdominal wall); alive on follow-up (~ 18 months after second treatment), no distant metastasis reported
[8] Loh et al. (2019)	47/F	Right lower abdomen (inguinal area)	Not reported	Not reported	Not performed	No nodal or distant metastasis on CT/PET at initial presentation	Poorly differentiated carcinoma	ER: positive, PR: weakly positive, Her2: negative	Initial complete surgical resection → Resection of recurrent mass → Adjuvant chemotherapy → Radiotherapy	Locoregional recurrence at 7 months; aggressive disease course; no distant metastasis reported
[9] Byon et al. (2021)	65/M	Left lower abdomen (suprapubic area)	Not reported	Not reported	Not performed	No nodal or distant metastasis on CT/PET at initial presentation	Invasive ductal carcinoma (grade 2)	ER and PR: positive, HER2: negative	Wide local excision → Adjuvant endocrine therapy (tamoxifen) → Subsequent systemic chemotherapy for metastasis	Recurrence at 3 years with distant metastasis (liver and pulmonary lymph nodes); progressive metastatic disease despite treatment.
[27] Bansal et al. (2022)	69/M	Left lower abdomen (inguinal area)	Not reported	To left inguinal lymphatic basin, based on imaging findings (axillary basins not assessed)	Not performed initially; after initial excision of tumor had lymph node dissection, which showed nodes negative (0/3)	No nodal or distant metastasis on CT/PET at initial presentation	Poorly differentiated invasive ductal carcinoma	ER and PR: positive, HER2: negative	Initial excision → revision wide local excision + lymph node dissection → Adjuvant radiotherapy → Hormonal therapy (tamoxifen)	No recurrence or metastasis; disease-free at 6 months
[25] Cicek et al. (2023)	67/F	Right lower abdomen (inguinal area)	Not reported	To right inguinal lymphatic basin, based on imaging findings (axillary basins not assessed)	Performed: SLN negative (two right inguinal SLN)	No nodal or distant metastasis on PET/CT; local vascular invasion (right common femoral vein)	Invasive ductal carcinoma	ER and PR: positive, HER2: negative	Neoadjuvant chemotherapy → Wide surgical excision with vascular resection and reconstruction + SLN excision → Adjuvant radiotherapy → endocrine therapy (letrozole)	No recurrence or metastasis; disease-free at 6 months
[26] Liu et al. (2024)	60s/F	Left upper abdomen (ipsilateral to prior orthotopic breast cancer)	Not reported	Not reported	Not performed	No nodal or distant metastasis on imaging workup; lesion represents cutaneous intraepidermal spread (Paget’s disease) rather than nodal metastasis	Mammary Paget’s disease with underlying DCIS	ER: positive, PR: negative, HER2: positive	Narrow-margin excision	No recurrence or distant metastasis on regular follow-up; abdominal EBC lesion occurred 4 years after initial breast cancer
Current case	64/F	Left upper abdomen	Performed using ICG fluorescence (single tracer, real-time intraoperative mapping)	Ipsilateral axillary drainage only; no inguinal drainage identified	Performed: SLN negative (one left axillary SLN)	No nodal or distant metastasis on imaging workup	Invasive papillary carcinoma (G2) with associated DCIS	ER and PR: positive, HER2: negative	Wide excision of ectopic breast tissue involving tumor → SLNB → Adjuvant endocrine therapy (letrozole)	No recurrence or distant metastasis; disease-free at 1-year follow-up

## Data Availability

Preparation of this report complied with the CARE guidelines. The data supporting the findings of this case report are available from the corresponding author upon reasonable request, in accordance with institutional and ethical regulations.
